# *N*-linked glycans: an underappreciated key determinant of T cell development, activation, and function

**DOI:** 10.1097/IN9.0000000000000035

**Published:** 2023-11-21

**Authors:** Mahmoud Abdelbary, Jeffrey C. Nolz

**Affiliations:** 1 Department of Molecular Microbiology and Immunology, Oregon Health & Science University, Portland, OR, USA; 2 Department of Cell, Developmental and Cancer Biology, Oregon Health & Science University, Portland, OR, USA; 3 Department of Dermatology, Oregon Health & Science University, Portland, OR, USA

**Keywords:** glycosylation, *N*-linked glycans, T cells, glycobiology, immune responses

## Abstract

N-linked glycosylation is a post-translational modification that results in the decoration of newly synthesized proteins with diverse types of oligosaccharides that originate from the amide group of the amino acid asparagine. The sequential and collective action of multiple glycosidases and glycosyltransferases are responsible for determining the overall size, composition, and location of *N*-linked glycans that become covalently linked to an asparagine during and after protein translation. A growing body of evidence supports the critical role of *N*-linked glycan synthesis in regulating many features of T cell biology, including thymocyte development and tolerance, as well as T cell activation and differentiation. Here, we provide an overview of how specific glycosidases and glycosyltransferases contribute to the generation of different types of *N*-linked glycans and how these post-translational modifications ultimately regulate multiple facets of T cell biology.

## 1. Introduction

Glycosylation is a post-translational modification that results in the decoration of proteins with an extensive array of oligosaccharides that orchestrate several cellular processes including protein folding, trafficking and secretion, stability, antigenicity, steric conformation, ligand-receptor interaction, and many other aspects of protein biology ^[1–4]^. Glycosylation can be broadly defined as being either *O*-linked or *N*-linked depending on whether the glycan chain originates from serine/threonine (S/T) or asparagine (N) amino acids, respectively ^[5–7]^.

The repertoire of glycans (both *O*- and *N*-linked) decorating multiple surface proteins on T cells is extremely diverse and both undergo continuous remodeling across all stages of T cell development and differentiation, thereby regulating many aspects of T cell biology including positive and negative selection in the thymus, immune tolerance, homeostatic proliferation, trafficking, antigen recognition, cytotoxicity, and cytokine responsiveness ^[6–11]^.

**Figure 1.**
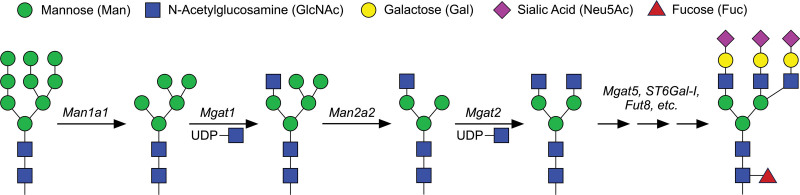
**Enzymatic synthesis of complex *N*-linked glycans occurs in a sequential fashion through the action of specific glycosidases and glycosyltransferases**. Trimming of terminal mannose residues through the action of *α*-mannosidase-I is required for hybrid *N*-linked glycan synthesis by the addition of GlcNAc by Mgat1. Further trimming of mannose by *α*-mannosidase-II and addition of GlcNAc by Mgat2 results in the precursor for the synthesis of complex *N*-linked glycans. The collective action of additional glycosyltransferases (Mgat5, Fut8, ST6Gal-I, etc) determines the extent of branching and complexity of the final *N*-linked glycan chain. The majority of complex *N*-linked glycans are decorated on one or more branches with extended repeats of *N*-acetyllactosamine, composed of *β*1,3-Gal-*β*1,4-GlcNAc, which are terminated following the capping of these repeats with an *N*-acetylneuraminic acid (Neu5Ac; Sialic acid) by ST6Gal-I. Fut8, fucosyltransferase 8; Man1a1, mannosidase-I; Man2a2, mannosidase-II; Mgat1, *N*-acetylglucosaminyltransferase-I; Mgat2, *N*-acetylglucosaminyltransferase-II; Mgat5, *N*-acetylglucosaminyltransferase-V; ST6Gal-I, *β*-Galactoside *α*2,6-sialyltransferase-I; UDP-GlcNAc, uridine diphosphate-GlcNAc.

**Figure 2.**
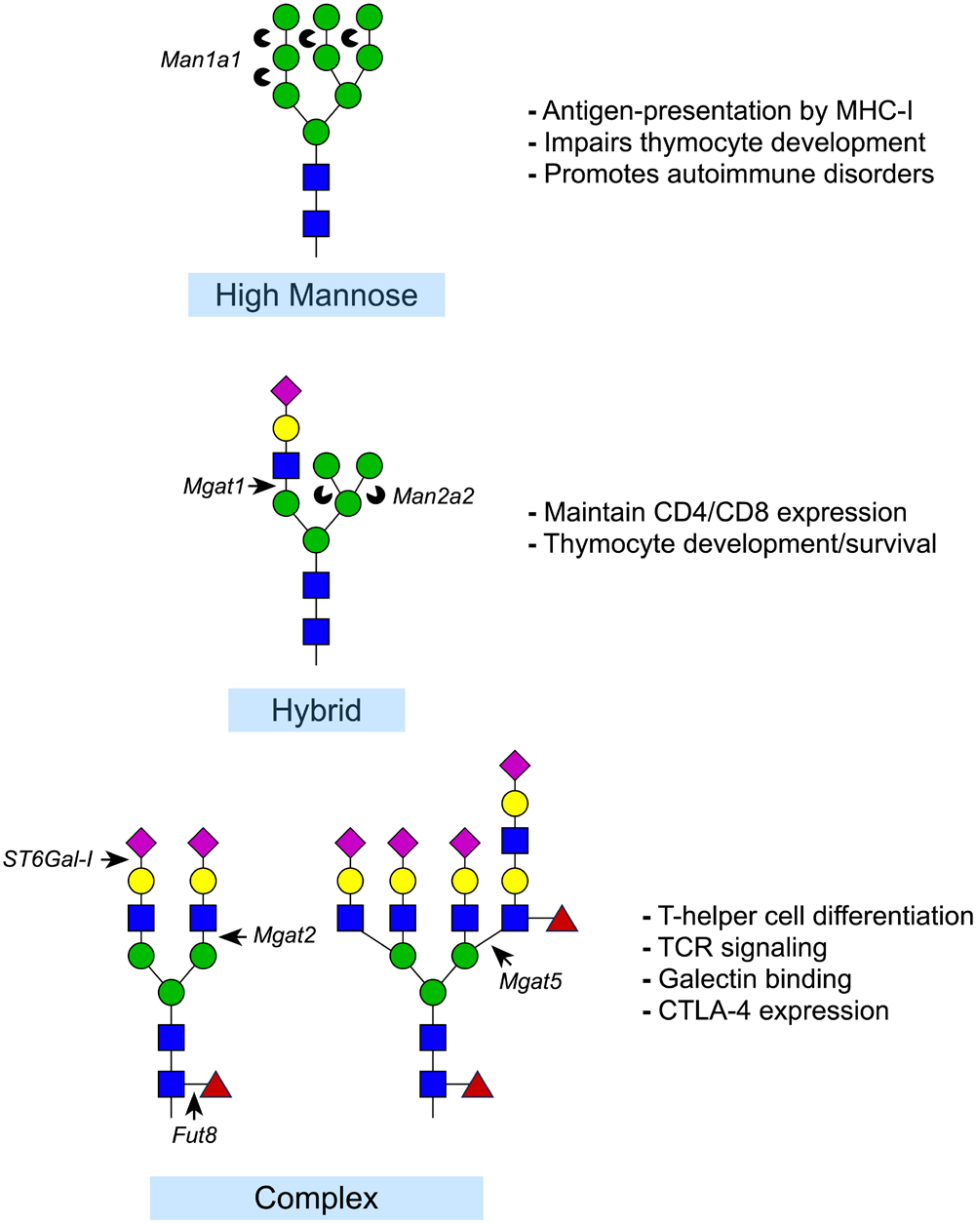
**Three major types of *N*-linked glycans and their influence on T cell biology**. In general, high-mannose, *N*-linked glycans are typically detrimental to T cells and severely impair thymocyte maturation leading to the development of various autoimmune diseases. Hybrid *N*-linked glycans containing terminal mannoses, but carrying a single branch are sufficient for normal development of thymocytes. Complex *N*-linked glycans can be highly diverse and regulate many aspects of T cell function and differentiation within the periphery to guard against hyperactivity, inflammation and autoimmunity.

The synthesis and maturation of *N*-linked glycans occur within the secretory pathway, beginning on the luminal surface of the endoplasmic reticulum where a 14-sugar oligosaccharide precursor is transferred from dolichol phosphate by the oligosaccharyltransferase complex that becomes covalently attached to asparagine located within the highly conserved N-X-S/T consensus motif, where X represents any amino acid except proline ^[12,13]^. Further branching and extension of the precursor *N*-linked glycan takes place as the nascent glycan proceeds throughout the Golgi network, which involves the collective and sequential action of several glycosidases, glycosyltransferases, and nucleotide-sugar substrates that together shape the final composition of the mature *N*-linked glycan (Figure 1). During maturation, the growing *N*-linked glycan passes through three structurally distinct forms (1) mannose rich form, (2) hybrid form with a single elongated antenna, and (3) complex form with bi-, tri-, or tetra-antennary structure (Figure 2). Trimming of terminal mannose residues by mannosidases and the subsequent initial extension of *N*-linked glycans by Mgat1 is critical for proper thymocyte survival and development. Once in the periphery, the extent of branching (by Mgat2 and Mgat5), as well as the overall complexity and composition of *N*-linked glycans (by multiple enzymes such as *β*-Galactoside *α*2,6-sialyltranferase-I [ST6Gal-I] and fucosyltransferase 8 [Fut8]) regulates many aspects of T cell biology including T cell receptor (TCR) clustering and activation threshold, interaction with carbohydrate-binding proteins (primarily galectins), as well as cytokine receptor stability and signaling. The detailed step-by-step process of *N*-linked glycan synthesis has been delineated in detail elsewhere ^[5,12–17]^. Here, we will focus our discussion on the contribution of the enzymes described above that function in the synthesis of *N*-linked glycans that have been shown to regulate various aspects of T cell biology.

## 2. Mannosidase-I and II

Mannosidase classes I and II (*Man1a1* and *Man2a2*) are two highly conserved eukaryotic Golgi enzymes that catalyze the cleavage of *α*1,2-, *α*1,3- or *α*1,6- glycosidic linkage of terminal mannose residues within the core of an oligosaccharide precursor of *N*-linked glycans ^[18–20]^ (Figures 1 and 2). Mannosidases are necessary for the synthesis of hybrid and complex types of *N*-linked glycans due to removal of the terminal mannose residues from the core allowing further processing and branching of the nascent *N*-glycan chain. The action of mannosidase-I upstream of Mgat1 leads to the formation of Man5GlcNAc2, which represents the building unit for the synthesis of branched (hybrid) *N*-linked glycan structures ^[19–21]^. The action of mannosidase-II downstream of Mgat1 further removes two mannose residues which can be further modified by Mgat2 to initiate the synthesis of complex *N*-linked glycans ^[22–24]^.

The surface expression of high-mannose *N*-linked glycans remains low across all thymocyte developmental stages, as quantified using either *Galanthus nivalis* lectin or concanavalin A plant-based fluorescent lectins, which display high affinity for *α*-mannose rich glycans ^[25,26]^. Elevated expression of high-mannose *N*-linked glycans has been linked to the pathogenesis of autoimmune diseases ^[7,27–29]^ and several mechanisms have been elucidated detailing the contribution of high-mannose *N*-glycans in exacerbating immune cell activation and immunopathology, such as excessive activation of the mannose-specific lectin receptors found on innate immune cells ^[7,27,28]^. Furthermore, this greater susceptibility to autoimmune diseases has also been attributed to diminished development of regulatory T cells (Tregs) concomitant with the accumulation of high-mannose *N*-glycans on thymocytes ^[25,30]^.

Even though complex *N*-linked glycans are the dominant form that decorates glycoproteins in mammalian cells, the *α* chain of MHC-II is reported to retain high-mannose *N*-linked glycans ^[8,31]^. Moreover, high-mannose *N*-linked glycans are essential for the proper folding of MHC-I in the ER as well as regulating proteasomal degradation of the *δ* subunit of CD3, suggesting that controlling the level of high-mannose *N*-glycans by mannosidases indirectly impacts T cell functions by modulating their capacity to interact with other cell types ^[19,32,33]^. Further investigation revealed that TCR engagement directly upregulated the gene expression of a wide variety of *N*-linked glycan-modifying enzymes including mannosidase-I and II enzymes, a phenomenon that was observed only in naïve T cells, since the expression level remained unchanged following restimulation of memory or effector T cells ^[34–36]^. Moreover, pharmacological inhibition of mannosidases via treatment with pyranose or furanose analogs such as 1-deoxymannjirimycin, kifunensine, or swainsonine leads to accumulation of mannose rich, unbranched *N*-linked glycans, which was accompanied by lower activation threshold of both murine and human naïve CD4^+^ T cells and enhanced skin-graft rejection mediated by alloreactive T cells ^[35,37]^. Alternatively, inhibition of mannosidase activity in human tumor cells enhanced immunogenicity and T-cell-mediated cytotoxicity ^[38]^. Inhibition of mannosidase-II also increased the adhesion of Jurkat T cells, causing them to form large cell aggregates due to enhanced surface expression of adhesion molecules such as CD54 and lymphocyte function-associated integrin 1 while reducing interaction with the carbohydrate-binding protein galectin-1, which preferentially binds to branched *N*-glycans, suggesting a potential role for mannosidase-II in modulating T cell interactions ^[39,40]^. Taken together, these data suggest an important contribution for mannosidases in guarding against aberrant activation of T cells.

## 3. *N*-acetylglucosaminyltranferases (GnTs/Mgats)

Branching of the nascent *N*-linked glycan chain is initiated in the medial Golgi through a family of enzymes known as *N*-acetylglucosaminyltranferases (GnTs), which are encoded by *Mgat* genes. Mgats catalyze the transfer of *N*-acetylglucosamine (GlcNAc) from uridine diphosphate-GlcNAc (UDP-GlcNAc) to either an *α* or *β* glycosidic linkage of an acceptor sugar residue ^[41]^. The degree of branching of *N*-linked glycans is dictated by the collective action of Mgats and ranges from hybrid glycans with a single antenna to complex *N*-glycans with bi-, tri-, or tetra-antennary structures (Figure 2). Dysregulated Mgat activity has been linked to multiple T-cell-mediated immunopathologies ^[42–47]^, while oral supplementation with the Mgat substrate GlcNAc elevated the level of *N*-glycan branching on T cells concomitant with mitigated inflammation, ameliorated severity of established autoimmunity and delayed onset of autoimmune disease, indicating the fundamental role for Mgats in regulating T cell activity ^[42,48,49]^. In this section, we will discuss three of the five well-characterized Mgat family members that are active participants in orchestrating T cell development and functions.

### 3.1 Mgat1 (GnT-I)

*N*-acetylglucosaminyltransferase-I, encoded by *Mgat1* gene, catalyzes the attachment of *β*1,2-GlcNAc to *α*1,3-linked mannose of the growing *N*-linked glycan core ^[50]^ (Figures 1 and 2). The action of Mgat1 represents a rate-limiting enzymatic step in the biosynthesis of hybrid and complex *N*-glycans, as it introduces the first branching antenna to the oligomannosidic *N*-glycan chain and genetic deletion of Mgat1 leads to total loss of branching on *N*-linked glycans ^[51–54]^. Mgat1 activity is essential for normal development, as evidenced by early death of mice embryos lacking Mgat1 approximately 10 days postnatal due to impaired tissue development ^[54–56]^.

In peripheral T cells, the differential gene expression of the various Mgats is tightly regulated by TCR signaling, as expression of Mgat1 and Mgat2 is reduced, while expression of Mgat5 is increased following stimulation with antibodies against CD3/CD28, suggesting that individual Mgats play distinct roles in regulating T cell activation and differentiation and may also suggest a limited requirement for Mgat1 in activated T cells ^[34]^. Furthermore, signaling downstream of the IL-7 receptor also increases expression of Mgat1 by ~twofold ^[57]^. Mgat1 has the highest affinity for UDP-GlcNAc, ~250fold higher than the lower affinity Mgat5 enzyme ^[34]^. Thus, diminished expression of Mgat1 in T cells following activation may increase the availability of UDP-GlcNAc to other Mgats (such as Mgat5) that are potentially more critical in regulating T cell effector functions.

*N*-linked glycan chain branching is essential for normal T cell development in the thymus. The extent of *N*-linked glycan branching oscillates across all stages of T cell development, reaching its lowest level on single positive thymocytes and peripheral T cells, which can be evaluated using the fluorescent plant lectins *Lycopersicon esculentum* lectin and *Phaseolus vulgaris* leucoagglutinin. Meanwhile, the synthesis of high-mannose *N*-linked glycans remained relatively constant across all stages of thymocyte development, suggesting that the extent of branching, rather than the overall abundance of *N*-linked glycans, is necessary for normal thymocyte development ^[25,26]^. The activity of Mgat1 appears to be temporally regulated, since genetic deletion of Mgat1 at the double negative stage 4, enhances apoptosis of the double positive thymocytes, thereby diminishing the total number of mature T cells that reach the periphery ^[25,26]^. In contrast, genetic deletion of Mgat1 in peripheral T cells did not alter their capacity to survive, suggesting that, unlike thymocytes, Mgat1-mediated branching of *N*-linked glycan is dispensable for the survival of peripheral T cells ^[26]^.

Further investigation of the mechanisms underlying Mgat1-mediated survival of thymocytes revealed that Mgat1 modulates the upper and lower TCR affinity threshold to both enhance positive selection and inhibit negative selection-associated thymocyte cell death. Mgat1 promotes positive selection of thymocytes by enhancing TCR sensitivity and signaling in response to low-affinity peptide-MHC complexes, mainly by increasing surface retention of CD4 and CD8 coreceptors and consequently inhibiting thymocyte death by neglect ^[25,26]^. Alternatively, Mgat1 inhibits negative selection of thymocytes by limiting TCR stimulation-mediated calcium influx and the subsequent cell death following the engagement of high-affinity peptide-MHC complexes and rather, promotes the development of Tregs ^[25,26]^. Taken together, these data indicate that *N*-linked glycans with at least a single antenna are essential to ensure proper T cell development within the thymus.

### 3.2 Mgat2 (GnT-II)

*N*-acetylglucosaminyltransferase-II, encoded by the *Mgat2* gene, functions downstream of mannosidase-II and catalyzes the attachment of *β*1,2-*N*-acetylglucosamine to the *α*1,6-linked mannose residue to form biantennary decorated *N*-linked glycans and initiate the synthesis of complex *N*-linked glycans ^[58,59]^ (Figures 1 and 2). Point mutations in Mgat2 have been identified in humans and are associated with congenital disorder of glycosylation IIa and general neurological defects, as well as increased risk of experimental autoimmune encephalomyelitis in mice ^[58,60–62]^. Mice lacking Mgat2 exhibit severe developmental defects in various tissues and die early postnatally, indicating the critical role of complex *N*-linked glycans in regulating normal development and physiology ^[50,61]^.

In contrast to Mgat1, genetic deletion of Mgat2 has only minimal impact on the surface expression of CD4 and CD8 ^[26]^. Furthermore, Mgat2 deficient thymocytes capable of synthesizing only hybrid type *N*-glycans developed normally in the thymus without changing the number of peripheral T cells ^[25,26]^. The lack of requirement for Mgat2-mediated branching of *N*-linked glycans by developing thymocytes could be explained through the observed compensatory increase of *N*-acetyl-lactosamine extension (extended repeats of *β*1,3-Gal-*β*1,4-GlcNac; LacNAc) on the single antenna of hybrid *N*-glycans. The majority of complex *N*-linked glycans are decorated with at least one or more antennas bearing LacNAc repeats, which serves as a ligand for the carbohydrate-binding proteins galectins, which are typically expressed by stromal cells of lymphoid tissues and function as endogenous ligands ^[63,64]^. Poly-LacNAc—galectin interactions lead to the formation of bulky structures, often referred to as galectin lattices, which are thought to regulate the spatial organization of various T cell surface proteins. The decoration of hybrid *N*-glycans with LacNAc repeats in Mgat2 deficient T cells may rescue the T cell interactions with other cells through enhancing galectin lattice formation within the thymus microenvironment, thereby maintaining the geometric distribution of T cell surface proteins and extend their retention or enhance signaling downstream of these surface receptors ^[58,65]^. Processing and presentation of carbohydrate bacterial-antigen, but not peptide antigens, by MHC-II also requires the expression of complex *N*-glycans as indicated by abolished T cell response to the polysaccharide antigens of commensal bacterial due to loss of antigen binding and presentation by MHC-II in Mgat2 deficient dendritic cells ^[66,67]^.

Sex differences in *N*-linked glycan branching have been reported in both aged mice and humans. Naïve CD4^+^ T cells from old females exhibited elevated basal levels of *N*-linked glycan branching, which was associated with reduced reactivity and proinflammatory cues when compared to either memory or old-male naïve CD4^+^ T cells ^[68]^. This sexual-dimorphic branching pattern has been attributed to elevated IL-7 signaling by naïve T cells from aged females, resulting in higher phosphorylation of the transcription factor STAT5 ^[68]^. Genetic deletion of Mgat2 enhanced TCR signaling and reactivity of old-female CD4^+^ T cells and Mgat2-deficient T cells exhibited greater capacity to eliminate salmonella infection due to greater T_H_17 differentiation when compared to wild-type CD4^+^ T cells from old females ^[68]^.

### 3.3 Mgat5 (GnT-V)

The contribution of *N*-acetylglucosaminyltransferase 5, which is encoded by the *Mgat5* gene, was first described in a mouse lymphoma cells line where it was found to be critical for the formation of tri- and tetra- antennary complex *N*-linked glycans by catalyzing the attachment of *β*1,6-GlcNac to an *α*1,6-linked mannose residue of the nascent *N*-glycan core as it passes through medial Golgi ^[69]^. Mgat5 mediated *β*1,6-GlcNAc decoration of *N*-linked glycans exhibits the highest binding affinity and avidity for galectins and has been reported to be heavily involved in regulating T-cell responses ^[26,70]^.

Early evidence reported upregulation of *Mgat5* gene expression following TCR stimulation, suggesting a role for *β*1,6-GlcNAc branching in regulating T cell activation ^[34,71]^. To further assess the functional contribution of Mgat5, *Mgat*5^−/−^ mice were generated and were reported to exhibit a lower threshold for T cell activation due to enhanced clustering and signaling of the TCR ^[70]^. Later evidence revealed that the galectin lattice formation due to interaction with the *β*1,6 attached poly-LacNAc *N*-linked glycans on the TCR forms a spatial barrier that restricts spontaneous clustering of TCR in the absence of T cell-specific antigen ^[7,70,72]^. Meanwhile, Mgat5-mediated branching of the *N*-glycans decorating the tyrosine phosphatase CD45 maintains its localization within the TCR microdomain, where it facilitates restriction of Lck activation and subsequent TCR signaling in resting T cells ^[73]^. In accordance with this, reduced antigen sensitivity of CD8^+^ T cells during chronic LCMV infection has been linked to IL-10-mediated expression of Mgat5 and binding of galectin-3 to the TCR ^[74]^. Furthermore, *Mgat5*^−/−^ mice exhibit greater susceptibility to multiple sclerosis (MS), experimental autoimmune encephalomyelitis (EAE) as well as the development of autoimmune-mediated glomerulonephritis ^[42,43,45,46,57]^. Similarly in humans, dysregulated Mgat5 activity has been linked to autoimmunity and hyperactivity of T cells. *Mgat5* gene polymorphism and reduced enzymatic activity in lymphocytes has been correlated to the severity and progression of MS ^[47,57,75,76]^. Moreover, in patients with ulcerative colitis, higher levels of *N*-glycan branching on TCRs and elevated *Mgat5* gene expression in mucosal (lamina propria) T cells strongly correlated with mild symptoms and enhanced responsiveness to standard therapy ^[77,78]^. Taken together, these data highlight the pivotal contribution of *β*1,6 branching of *N*-linked glycan in guarding against aberrant T cell activation and autoimmune disease development.

In addition to regulating TCR clustering and signaling, further studies revealed that Mgat5 is also critical to maintaining surface expression/retention of the inhibitory receptor CTLA-4 on T cells due to enhanced CTLA-4-galectin lattice formation ^[34,79]^. Mgat5 deficiency further contributes to aggravating autoimmunity by favoring the differentiation of the more proinflammatory T_H_1 and T_H_17 over T_H_2 CD4^+^ T cells ^[49,80]^. On the other hand, mice given oral supplements of GlcNAc or overexpression of Mgat5 increased regulatory T cell differentiation and maintenance due to enhanced surface retention of the high-affinity *α* chain of IL-2 receptor (CD25) concomitant with reduced T_H_1 and T_H_17 responses ^[49,81]^. In contrast to Mgat1, Mgat5 seems to contribute only minimally to either positive or negative selection of thymocytes ^[26,43]^. Taken together, these data provide strong evidence that Mgat5 plays a critical role in modulating T cell activation to ensure optimal T cell responses and to avoid exceeding the autoimmunity threshold.

## 4. Fucosyltransferase 8

**Figure 3.**
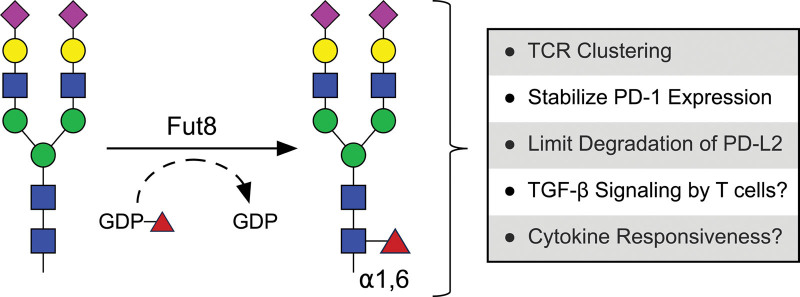
**Core fucosylation of *N*-linked glycans regulates multiple facets of T cell biology**. Fucosyltransferase 8 (Fut8) catalyzes the addition of an a1,6 linkage fucose to the innermost *N*-acetylglucosamine residue of *N*-linked glycans, a process known as core fucosylation. Core fucosylation of *N*-linked glycans influences T cell activity and function through a variety of known and proposed mechanisms.

The Golgi-localized Fut8 is the only enzyme in mammals, among the 13 fucosyltransferases, that catalyzes *α*1,6- attachment of L-fucose to the innermost asparagine-linked GlcNAc, forming what is widely described as core fucosylation (Figure 3) ^[82–84]^. Loss of Fut8 enzymatic activity has been reported in humans and is associated with severe developmental defects along with respiratory and neurological complications ^[85]^. Furthermore, ~80% of *Fut8*^−/−^ mice die within 3 days of birth and the surviving *Fut8*^−/−^ mice exhibited severe growth retardation along with renal and respiratory impairments. Further investigations revealed that these physiological defects are largely due to diminished signaling downstream of multiple growth factor receptors, TGF-*β* receptor, and *α*_3_*β*_1_ integrin, along with elevated matrix metalloproteinases activity within the affected organs, demonstrating that core fucosylation of *N*-linked glycans is critical for normal organogenesis and physiology ^[86–91]^.

Core fucosylated glycoproteins present on the surface of a variety of immune cells have been reported and a growing body of evidence suggests a role for core fucosylation in regulating immune responses. T cells isolated from inflamed intestinal mucosal tissues exhibited elevated levels of core fucosylated glycoproteins in both humans and mice with colitis, systemic lupus erythematosus, or EAE ^[92,93]^. Genetic deletion of *Fut8* ameliorated autoimmune-associated inflammation in mice, which was associated with decreased inflammatory cytokine production by CD4^+^ T cells ^[92,93]^. Another study found that global absence of Fut8 impaired CD4^+^ T cell-B cell interactions without affecting the interaction between CD4^+^ T cells and dendritic cells, which was associated with diminished humoral responses following infection with *Salmonella typhimurium* ^[94]^. However, it is important to note that these studies were performed using mice with complete and global Fut8 deficiency, which as mentioned earlier, exhibit broad and severe developmental defects, making it difficult to conclude that the overall dysregulated immune response is solely due to aberrant T cell functions.

TCR activation triggers transient upregulation of the inhibitory receptor PD-1, which functions to suppress T cell activity, however, the surface expression of PD-1 persists under conditions of continuous antigen-mediated TCR stimulation, which contributes to progressive loss of T cell effector functions ^[95–98]^. PD-1 is a highly glycosylated surface protein and core fucosylation of its *N*-linked glycans (specifically those attached to Asn49 and Asn74) are essential to stabilize PD-1 surface expression on T cells, as well as PD-1 recruitment to TCR microclusters upon engaging its ligand PD-L1 ^[99]^. In vitro work performed in Jurkat T cells revealed that core fucosylation protected PD-1 from ubiquitination and subsequent proteasomal degradation ^[100]^. Similarly, core fucosylation also guards against ubiquitination and degradation of PD-L2 (a PD-1 ligand). Substitution of Arg168, Glu171, and Tyr174 of PD-L2 with Ala disrupted its interaction with Fut8 and triggered T-cell-mediated antitumor response due to degradation and absence of PD-L2 on squamous cell carcinoma tumor model ^[101]^. In line with these data, both genetic deletion and pharmacological inhibition of Fut8 enhanced T cell proliferation, effector functions, and antitumor immunity in mice ^[99,100]^. Meanwhile, enhanced core fucosylation of MHC-II in both mice and humans increased its surface stability on melanoma cells leading to greater abundance of tumor-infiltrating T cells, enhanced antitumor T-cell-mediated immunity, and limited progression of melanoma ^[102]^. Taken together, these studies provide evidence that core fucosylation of *N*-linked glycans is involved in shaping the repertoire of cell surface proteins which alters T cell functions and their ability to eradicate infections and tumors.

Core fucosylated *N*-linked glycans have also been observed on other surface glycoproteins that are critical for regulating various aspects of T cell responses, however, further studies are needed to directly assess whether core fucosylation of these glycoproteins contributes to conformational regulation, proper protein folding, surface expression, signal transduction or receptor-ligand interactions. For example, IL-21 is critical for promoting immunity through suppressing regulatory T cell activity and enhancing CD8^+^ T cell proliferation, maintenance, effector functions, and responsiveness to immunotherapy during chronic viral infections and cancer ^[103–108]^. Within the IL-21 receptor, an *N*-linked glycan bearing a core fucose has been observed on Asn54 and is necessary to stabilize the expression of the IL-21 receptor when expressed in 293T cells, however, whether core fucosylation stabilizes expression of the IL-21 receptor remains to be confirmed in vivo ^[109]^. In addition, the crystal structure of the heterotrimeric IL-2 receptor complex revealed an *α*1,6 core fucose on the *N*-linked glycan synthesized on Asn17 of the IL-2Rβ chain. This fucose formed 4 hydrogen bonds with Arg105 and Leu106 on the D2 domain of IL-2Rβ ^[110]^, which may help stabilize the spatial orientation of the D1 and D2 domains which are necessary for ligand binding (IL-2 and IL-15) ^[110]^. Finally, it is well established that TGF-*β*1 regulates many aspects of T cell differentiation and function as documented in several studies, therefore, it is highly likely that diminished signaling downstream of the TGF-*β*1 receptor will enhance T cell activity, since lack of core fucosylated *N*-linked glycans on the TGF-*β*1 receptor was found to negatively regulate its function, however, this remains to be further investigated ^[86]^.

## 5. *β*-Galactoside *α*2,6-sialyltranferase-I

ST6Gal-I preferentially modifies complex *N*-linked glycans by catalyzing attachment of a terminal *α*2,6-*N*-acetylneuraminic acid (sialic acid) to *N*-acetyl-polylactosamine branches *(Galβ*1,4-GlcNac) and blocking further extension ^[111–113]^. *α*2,6-sialic acid capping of *N*-linked glycans on CD45 is critical to inhibit galectin-1-induced cell death of mature medullary thymocytes and peripheral naïve T cells primarily through enhancing the phosphatase activity of CD45 ^[114–117]^. Furthermore, T cell activation suppresses ST6Gal-I activity and expression of ST6Gal-I is reduced on recently activated effector and memory T cells compared to naïve T cells ^[34,118,119]^. The differential expression of ST6Gal-I also regulates the balance between subsets of helper CD4^+^ T cells. For example, T_H_2 CD4^+^ T cell subsets were protected from galectin-1-induced cell death due to greater *α*2,6 sialylation of their surface *N*-linked glycans compared to T_H_1 and T_H_17 T cells ^[120]^. In agreement with this observation, *ST6Gal-I*^−/−^ mice were more prone to autoimmune disease due to increased abundance and enhanced responsiveness of T_H_1 and T_H_17 CD4^+^ T cells ^[120]^. Later evidence linked high levels of ST6Gal-I expression and activity to the expression of the transcription factor TCF-1 by a pathogenic population of CD4^+^ T cells that chronically produced IFN-γ and in turn, promoted intestinal inflammation ^[121–123]^. Taken together, these findings highlight the pivotal role of *α*2,6 sialylation of *N*-linked glycan in regulating the differentiation of CD4^+^ T cells. Although CD8^+^ T cells appear to develop normally in the absence of ST6Gal-I ^[113]^, comprehensive evidence investigating the functional contribution of ST6Gal-I in CD8^+^ T cells is still lacking. In one study, the primary expansion of antigen-specific CD8^+^ T cells and the differentiation of terminal effectors were both impaired in the absence of ST6Gal-I and that was speculated to be due to dysregulated IL-2 signaling ^[113]^. In contrast, another study observed normal kinetics and effector functions of influenza-specific CD8^+^ T cells in *ST6Gal-I*^−/−^ mice ^[124]^. Future studies will be needed to address these opposing findings and to determine the role(s) of ST6Gal-I in regulating CD8^+^ T cell responses.

## 6. Conclusions and future perspectives

As discussed in this review, *N*-linked glycan synthesis involves the collective action of an extensive array of glycan-modifying enzymes that eventually dictates the final composition and function of the glycan structures decorating the surface proteins of T cells. The activity of various members of the *N*-linked glycan synthesis machinery is highly regulated across various stages of T cell development and differentiation. The presence of branched *N*-linked glycans on the surface of T cells is essential to ensure proper T cell development and formation of a diverse pool of naïve T cells primarily by altering TCR affinity limits to enhance thymocyte survival during selection. Following the seeding of secondary lymphoid tissues in the periphery, the interaction of branched *N*-linked glycans with various galectins promotes survival and inhibits spontaneous activation of T cells by controlling the spatial distribution of components of the TCR signaling complex. We also discussed how various modifications of *N*-linked glycans regulate surface retention or inhibit the degradation of certain proteins that directly affect T cell activation, cytokine responsiveness, and differentiation.

Overall, these findings highlight fundamental principles that must be considered regarding autoimmune disease treatment, vaccinations, and immunotherapy approaches. Recent treatment strategies are often strongly focused on either enhancing or limiting T cell responses at the epigenetic and/or transcriptional level, without considering that these approaches may also dramatically remodel the glycan repertoire decorating numerous surface receptors. The extent of *N*-linked glycan branching, as well as the composition of each antenna, contribute to the overall quality and strength of the T cell response. Thus, therapeutically shaping the nature and extent of *N*-linked glycan synthesis by antigen-specific T cells represents a largely unexplored opportunity for the potential development of novel disease treatments that could have major implications for either enhancing or limiting T cell responses in vivo.

## Conflicts of interest

The authors declare that there are no conflicts of interest.

## Funding

Research in the Nolz Laboratory is supported by grants from the National Institutes of Health (R01-AI132404, R01-AI143664, R21-AI159401, and R21-AI173440).

## Acknowledgments

The authors would like to thank all current and former members of the laboratory for helpful discussion and their important contributions to this area of investigation.
